# Storage-associated physicochemical changes in extracellular vesicles derived from human mesenchymal stromal cells

**DOI:** 10.3389/fbioe.2026.1829175

**Published:** 2026-07-30

**Authors:** Inna Gordiienko, Nadiia Petryk, Ivan Kushka, Olha Nesenyuk, Marta Lukasevych, Svitlana Novikova, Igor Zlatskiy, Alona Zlatska

**Affiliations:** 1 Biotechnology Laboratory, Medical Company “Good Cells”, Kyiv, Ukraine; 2 R.E. Kavetsky Institute of Experimental Pathology, Oncology and Radiobiology NAS of Ukraine, Kyiv, Ukraine; 3 Institute of Genetic and Regenerative Medicine, M. D. Strazhesko National Scientific Center of Cardiology, Clinical and Regenerative Medicine, National Academy of Medical Sciences of Ukraine, Kyiv, Ukraine

**Keywords:** extracellular vesicles, human mesenchymal stromal cells, physicochemical properties, storage conditions, storage temperature

## Abstract

**Introduction:**

Extracellular vesicles (EVs) derived from mesenchymal stromal cells (MSCs) are considered a promising therapeutic tool due to their involvement in intercellular signaling and their ability to carry a wide range of biologically active molecules, including proteins, lipids, and nucleic acids. Standardized protocols for EV manufacturing and evaluation of EV properties under different storage conditions are important to ensure the quality, reproducibility, safety, and therapeutic efficacy of EV-based products. This study evaluated the physicochemical stability of EVs derived from MSCs of various tissue origins under different storage temperatures.

**Materials and Methods:**

MSC populations were isolated from adipose tissue (ADSCs), umbilical cord (UC-MSCs), and dental pulp (DPSCs). The isolated cells met the minimal criteria for defining MSCs. EVs were isolated from conditioned media by differential ultracentrifugation and characterized by nanoparticle tracking analysis (NTA) and ELISA detection of the EV-specific markers CD63, CD81, and TSG101. Total protein concentration was determined using the Pierce BCA colorimetric assay. The physicochemical stability of MSC-derived EVs diluted in Hank’s solution was evaluated at predefined intervals during storage at +4 °C to +6 °C, –20 °C, and –80 °C.

**Results:**

EV particle concentration and total protein concentration remained stable for at least 14 days at +4 °C to +6 °C and for at least 6 months at –20 °C. EV samples preserved their characteristics for at least 10 months when stored at –80 °C. The assessment of bFGF complemented conventional physicochemical characterization by providing information on the preservation of a selected biologically relevant quality attribute of EV preparations.

**Conclusion:**

MSC-derived EVs diluted in saline solution retain physicochemical stability during long-term storage under clinically relevant conditions, supporting the further development of EV-based therapeutic products.

## 1 Introduction

Extracellular vesicles (EVs) derived from mesenchymal stem cells (MSCs) are considered promising instrument of cell-free therapeutic approaches due to their involvement in intercellular communication and their ability to transfer biologically active molecules, including proteins, lipids, and nucleic acids (Wright et al., 2021; Soliman et al., 2021; Krampera and Le Blanc, 2021; Zhuang et al., 2021; Wang et al., 2024). Increasing evidence suggests that MSC-derived EVs may contribute to tissue repair, immunomodulation, and regulation of inflammatory processes, making them attractive candidates for regenerative medicine applications (Gurung et al., 2021; Hade et al., 2021; Gordiienko et al., 2023; Tan et al., 2024).

In addition to their therapeutic potential, EVs are being actively investigated as biomarkers for various pathological conditions, including cardiovascular diseases, cancer progression, and pregnancy-related disorders (Kalluri and LeBleu, 2020; Ratajczak et al., 2006; Valadi et al., 2007). Furthermore, EVs are explored as potential drug delivery systems because of their biological compatibility and ability to mediate targeted intercellular signaling (Bruno et al., 2009; Van Niel et al., 2018; Skotland et al., 2020). However, despite the growing number of experimental and clinical studies involving EV-based products, the standardization of EV manufacturing, characterization, quality control, and storage conditions remains a significant challenge (Harrell et al., 2022; Huang et al., 2026; Muthu et al., 2021; Coumans et al., 2017; Fernández-Santo et al., 2022).

One of the important but insufficiently studied aspects of EV standardization is the preservation of EV physicochemical properties during storage. Storage conditions may affect EV particle concentration, size distribution, protein composition, membrane integrity, and biological activity, potentially influencing the reproducibility and translational applicability of EV-based products (Panda et al., 2021; Soni et al., 2021; Almeria et al., 2022; Shekari et al., 2023). In addition, EVs derived from MSCs of different tissue origins may exhibit distinct biological and biochemical characteristics, which could influence their stability profiles during storage. Establishing reproducible storage conditions is essential for batch-to-batch consistency, product release criteria, transportation, and long-term storage of EV-based medicinal products.

Therefore, the aim of this study was to evaluate the physicochemical stability of EVs derived from mesenchymal stem cells of different tissue origins under various temperature storage conditions.

## 2 Materials and methods

### 2.1 Ethics and consent

The experiments with the use of human cell culture *in vitro* were carried out in accordance with the human experiment issues of the Code of Ethics of the World Medical Association (Declaration of Helsinki). In all cases, voluntary informed consent was signed by MSC donors in accordance with the laws of Ukraine. The study protocol (No. 1/06-23 from 1 June 2023) was approved by the Bioethics commission of the SI National Scientific Center named after M. D. Strazhesko NAMS of Ukraine. The cell culture was carried out at the biotechnological laboratory Medical Company “Good Cells” (Kyiv, Ukraine) (License to operate the banks of human cord blood, other tissues and cells; issued by the Ministry of Health of Ukraine AE No. 2088 from 11.09.2020 and No. 2214 from 29.09.2020).

MSC samples (n = 6) were obtained from various tissues of healthy donors (3 females and three males) with normal somatometric and biochemical parameters without signs of obesity or viral or microbial infection. The age of the patients was 24 ± 4.0 years. The body mass index (BMI) of the adipose tissue donors was 21 ± 1.4.

MSC populations were isolated from adipose tissue (ADSCs), the umbilical cord (UC-MSCs), and dental pulp (DPSCs). The experimental design is shown in Figure 1.

**FIGURE 1:**
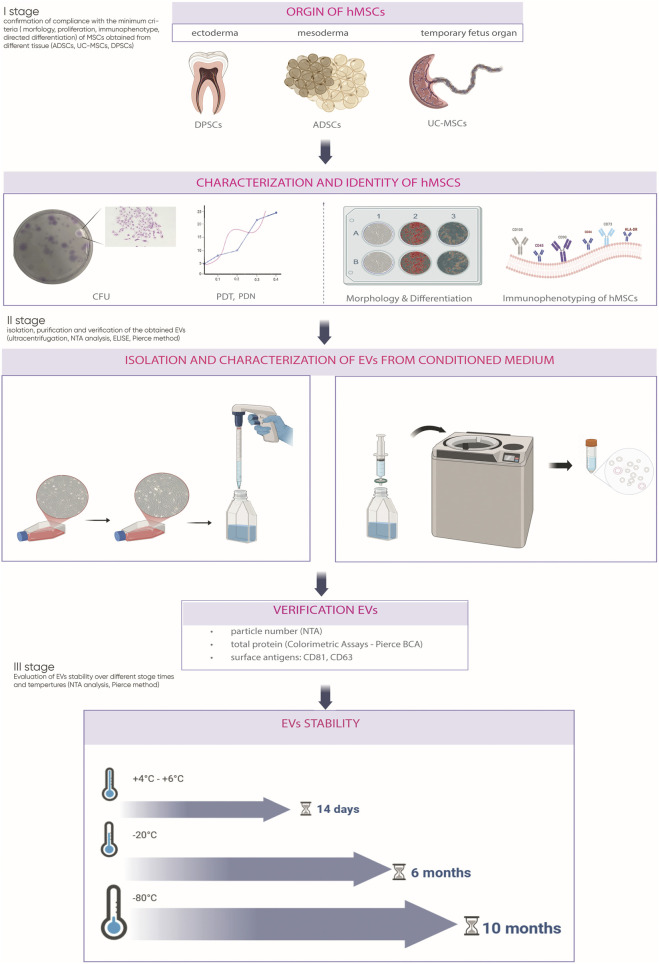
Experimental design. I stage - Confirmation of compliance with the minimum criteria (morphology, proliferation, immunophenotype, directed differentiation) of MSCs obtained from different tissue (ADSCs, UC-MSCs, DPSCs). II stage - Isolation, purification and verification of the obtained EVs (ultracentrifugation, NTA analysis, ELISA, Pierce method). IIІ stage - Evaluation of EVs stability over different storage times and temperatures (NTA analysis, Pierce method).

### 2.2 Cell isolation and culture

MSCs were isolated as previously described (Gordiienko et al., 2025; Zlatska et al., 2020; Zlatska et al., 2018; Gubar et al., 2017; Zlatskiy et al., 2020). In brief, UC-MSCs and ADSCs were isolated from tissues through enzymatic digestion with 0.1% collagenase and 0.1% pronase in 2% fetal bovine serum (FBS) (all from Sigma‒Aldrich, United States) for 1 h at 37 °C with constant agitation. All the debris and connective tissue were separated from the cells via centrifugation (600 × g, 5 min). After being washed, the cells were cultivated in growth medium containing basal α-MEM supplemented with 10% FBS, 2 mM L-glutamine, 1% antibiotic-antimycotic solution, and 1 ng/mL bFGF (all from Sigma‒Aldrich, United States). The cells were cultured in a CB210 multigas incubator (Binder, Germany) at 37 °C in a humidified atmosphere with 5% CO_2_ and 5% O_2_.

DPSCs were also isolated through the enzymatic digestion described above. After being washed, the DPSCs were cultured in T25 flasks coated with gelatin in the following growth media: basal α-MEM, 5% FBS, 1% IST supplement (Gibco, United Kingdom), 2 mM L-glutamine, 5 ng/mL bFGF, 5 ng/mL EGF (Sigma‒Aldrich, United States), and 1% antibiotic/antimycotic solution.

### 2.3 Colony-forming unit assay

A detailed description of the method was presented in previous studies (Zlatska et al., 2020; Zlatska et al., 2018). To assess clonogenic potential, cells were seeded at a density of 100 cells per 100 mm gelatin-coated Petri dish (SPL, Korea) in growth medium (as described above) supplemented with 20% FBS and cultured for 14 days. Colony formation efficiency (or plating efficiency, PE) was calculated according to the standard formula (Freshney, 2010):

$$\text{PE},\%=\left(\text{no}.\text{ of colonies counted}/\text{no}.\text{ of cells inoculated}\right)\times 100$$

The cell population doubling time (PDT) were calculated according to the following standard formula (Freshney, 2010):

$$\text{PDT}=T\;/\;3.31\times \mathrm{lg}\left(X_{k}/\;X_{0}\right)$$

where X_k_ is the number of obtained cells; X_0_ is the number of plated cells; T is the cell culture time.

### 2.4 Flow cytometry analysis of cell surface and intracellular marker expression

A detailed description of the method was presented in previous studies (Gordiienko et al., 2025; Zlatska et al., 2020; Zlatska et al., 2018; Gubar et al., 2017; Zlatskiy et al., 2020). Flow cytometry was performed for all the obtained populations of MSCs. To determine the expression of cell surface markers, cell suspensions (1 × 10^6^ cells) were incubated with fluorescein isothiocyanate (FITC), phycoerythrin (PE) or peridinin chlorophyll protein-cyanine5.5 (PC-5.5)-conjugated monoclonal antibodies specific for human mesenchymal and hematopoietic lineage markers at room temperature in the dark for 30 min (all BD Bioscience, United States). The selected antibodies were against CD73, CD90, CD105, CD34, CD45 and HLA-DR (all BD Bioscience, United States). Additionally, we tested DPSCs to determine the presence of the nestin antibody. The samples were sorted on a CytExpert flow cytometer (Beckman Coulter, United States). The obtained data were analyzed via CellQuest software (BD Bioscience, United States).

### 2.5 Directed multilineage differentiation assay

The protocol of the directed multilineage differentiation assay was described previously in detail (Gordiienko et al., 2025; Zlatska et al., 2020; Zlatska et al., 2018; Gubar et al., 2017; Zlatskiy et al., 2020). Briefly, cells were seeded on a 6-well plate (SPL, Korea) at a seeding density of 50 × 10^3^ cells per well. After 24 h of cultivation, the quality of the culture was assessed on the basis of its ability to adhere to plastic and its fibroblast-like morphology. The growth medium was changed every 2–3 days until the culture reached 90% confluency. After this, the medium was replaced either with growth medium or with induction medium for directed differentiation.

Osteogenic differentiation was induced by culture in basal α-MEM supplemented with 10% FBS, 100 nM dexamethasone, 10 mM β-glycerophosphate, 50 μg/mL ascorbate-2-phosphate (all from Sigma, United States) and 1% antibiotic/antimycotic. Adipogenic differentiation was induced by culture in high-glucose DMEM (4.5 g/L) (BioWest, France) supplemented with 10% FBS, 1 μM dexamethasone, 200 μM indomethacin, 500 μM isobutylmethylxanthine, 5 μg/mL insulin (all from Sigma‒Aldrich, United States), 5% horse serum (BioWest, United States) and 1% antibiotic/antimycotic solution.

The medium was changed every 2 days for 21 days. Adipogenic and osteogenic differentiation assays of DPSCs were carried out on a collagen substrate.

### 2.6 Cytochemical detection

A detailed description of the method was presented in previous studies (Zlatska et al., 2020; Zlatska et al., 2018). For CFU staining, the cell colonies were fixed for 20 min with 96% ethanol, washed with PBS, and stained with azure-eosin via the Romanowsky-Giemsa method (all: Makrokhem, Ukraine) for 20 min. To confirm osteogenic and adipogenic differentiation, the cells were fixed for 20 min in 4% formalin (Makrokhem, Ukraine), washed with PBS (Sigma-Aldrich, United States), and stained for 20 min with a 2% solution of Alizarin Red S (pH 4.1; for detecting calcified extracellular matrix deposits) or a 0.5% solution of Oil Red O (for staining of neutral lipids) (all–Sigma-Aldrich, United States).

### 2.7 Microscopy

MSC cultures were visualized and photographed via a Carl Zeiss Axio ObserverA1 microscope, an Axio Cam ERc 5 s camera, and ZEN 2012 software (Carl Zeiss, Germany). The cell cultures were photographed with phase contrast during the cultivation of the cells at points of change in the culture medium. Assessment of the level of cytochemical detection of the efficiency of adipo- and osteogenic differentiation was carried out via visual control in transmitted light.

### 2.8 Isolation of EVs

A detailed description of the method was presented in previous studies (Gordiienko et al., 2025). When the cell cultures reached 70% confluency, they were washed with phosphate-buffered saline (PBS) to eliminate residual serum and cellular debris. Subsequently, 25 mL of induction medium without phenol red (Gibco, United States) and devoid of xenogenic serum and exogenous growth factors was added. The MSCs were cultured for an additional 48 h. The conditioned medium, containing secreted growth factors and EVs, was carefully collected and subjected to centrifugation at 3,000 *g* for 15 min to remove residual cell debris and apoptotic bodies. An Eppendorf 5810R centrifuge was used (Eppendorf, Hamburg, Germany). Following centrifugation, the supernatant was filtered through a 0.22 μm filter (Sarstedt, Germany) to ensure the removal of any remaining larger particles. To further purify the EVs, the filtered medium was ultracentrifuged at 100,000 × g for 90 min (Ultracentrifuge–Avanti JXN-30, Beckman Coulter, United States). The pellet, containing the EVs, is then resuspended in Hank’s solution (Biowest, France) for downstream applications. EV pellets isolated from MSCs of each type obtained from three donors were pooled and then proceeded to further analysis. The isolated EVs were characterized via nanoparticle tracking analysis (NTA) and the Pierce protein assay as described below to confirm their identity and purity. The obtained EV preparations were aliquoted into 2 mL portions at a concentration of 1 × 10^9^ particles/mL. All aliquots were intended for single use, and repeated freeze–thaw cycles were avoided.

### 2.9 Nanoparticle tracking analysis

A detailed description of the method was presented in previous studies (Gordiienko et al., 2025). Nanoparticle tracking analysis (NTA) was applied to determine the particle size and concentration of all the samples via a NanoSight LM10 instrument (Malvern Instruments Ltd. (United Kingdom)) equipped with NTA 3.0 analytical software and an additional 488 nm laser. The samples were diluted in Hank’s solution (Biowest, France) to an appropriate concentration before analysis. At least five 60 s videos were recorded per sample in light scatter mode with a camera level of 13–16. The software settings were kept constant for all measurements of EVs (screen gain 3–10, detection threshold 2–5).

### 2.10 Protein quantification

A detailed description of the method was presented in previous studies (Gordiienko et al., 2025). The protein concentration in the EV samples was measured via a Pierce BCA protein assay kit (Thermo Fisher Scientific, United States). The assay was performed according to the manufacturer’s instructions in the working range of 5–250 μg/mL via a standard protocol.

### 2.11 Electron microscopy of whole-mounted EVs

EV samples were prepared for ultrastructural analysis using a whole-mount staining and embedding procedure. Briefly, concentrated EV suspensions were fixed by mixing with an equal volume of 4% formaldehyde (Sigma-Aldrich, cat. No. P6148) to achieve a final concentration of 2% formaldehyde. Aliquots (5 µL) of the fixed suspension were deposited onto Formvar-carbon-coated copper electron microscopy grids (EMS, cat. No. FCF2010-Cu-SB) and allowed to adsorb for 20 min at room temperature. The grids were washed on droplets of phosphate-buffered saline. The samples were then post-fixed on 50-µL droplets of 1% glutaraldehyde (Sigma-Aldrich, cat. No. G5882) for 5 min, followed by eight sequential washes on 100-µL droplets of distilled water. For contrasting and embedding, the grids were transferred directly onto an ice-cold mixture of 4% uranyl acetate (SPI Supplies, cat. No. 02624-AB) and 2% methyl cellulose (1:9 ratio, vol/vol) and incubated for 10 min on ice. Excess fluid was carefully blotted using filter paper to leave a uniformly thin methyl-cellulose film over the EV layer. The grids were air-dried for 5–10 min before imaging with a JEM-100CX (JEOL) transmission electron microscope operating at an accelerating voltage of 80 kV.

### 2.12 ELISA immunoassay

ELISA kits were used to detection of bFGF (Sigma-Aldrich, Merck, Germany) CD63, CD81, TSG101 and Calnexin (all Novus Biologicals, United States) in EVs samples. Assays were performed according to the manufacturer’s instructions. The plates were read on a Multiskan SkyHigh Microplate Spectrophotometer (Thermo Fisher Scientific, United States).

### 2.13 Statistics

A sample of n ≥ 3 was used to analyze the results. Data are presented as mean and standard deviation (M± SD). Statistical analyses were performed via one-way analysis of variance (ANOVA) via Origin Pro software and Microsoft Excel software. Differences were considered to be statistically significant when p < 0.05.

## 3 Results

### 3.1 Isolation of primary cell populations from lipoaspirate, umbilical cord, and human dental pulp: Morphological characterization of cultured cells

To confirm that the isolated cell populations met the minimal criteria for human mesenchymal stromal cells (Dominici et al., 2006), phase-contrast microscopy was performed to evaluate their morphology (Figure 2).

**FIGURE 2:**
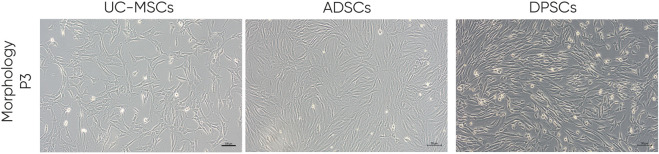
Representative morphology of UC-MSCs, ADSCs and DPSCs at passage 3 (P3). Phase-contrast microscopy. Scale bar = 100 µm.

Cells isolated from lipoaspirate (ADSCs) demonstrated adherence to plastic culture surfaces and exhibited elongated fibroblast-like morphology with uniform distribution throughout the culture area. Umbilical cord-derived MSCs (UC-MSCs) formed small colonies of adherent fibroblast-like cells with less homogeneous distribution during the initial stages of culture. Dental pulp-derived MSCs (DPSCs), isolated using enzymatic digestion, showed stable adherence to plastic surfaces and displayed spindle-shaped to stellate morphology with pronounced proliferative activity.

### 3.2 Determination of proliferative activity and assessment of colony formation efficiency

Proliferative activity and colony-forming ability are important characteristics used for the assessment of MSCs *in vitro*.

The proliferative activity and colony-forming efficiency of UC-MSCs, ADSCs, and DPSCs were evaluated at passages 1–3 (P1–P3) for each donor in triplicate. A summary of the colony-forming efficiency data is presented in Table 1.

**TABLE 1:** Colony-forming efficiency of different MSC populations at passages 1–3 (P1–P3) (n = 3, mean ± SD).

MSCs type	Р1	Р2	Р3
ADSCs	33.0% ± 0.6%	26.9% ± 1.9%	20.6% ± 2.5%
UC-MSCs	39.1% ± 1.4%	32.8% ± 0.5%	20.6% ± 2.2%
DPSCs	43.1% ± 1.0%	38.8% ± 1.7%	27.6% ± 0.8%

The dynamics of colony-forming efficiency showed a similar pattern across all studies cell types, with a characteristic peak at P1 and following reducing at P2-P3. Among the investigated MSC populations, DPSCs demonstrated the highest colony-forming efficiency at P3, reaching, reaching 27.6% ± 0.8%. These findings confirm the ability of UC-MSCs, ADSCs, and DPSCs to form colonies under clonal density conditions, consistent with one of the characteristic *in vitro* properties of MSCs that reflect proliferation activity and stemness.

Proliferative activity was additionally evaluated by assessing population doubling time (PDT) in all MSC types. The analysis was performed at P1, P2, and P3 for each donor in triplicate. A summary of the PDT values is presented in Table 2.

**TABLE 2:** Summary of PDT for different types of MSCs at passages 1–3 (P1-P3) (n = 3, M ± SD).

MSCs type	Р1	Р2	Р3
ADSCs	30.6 ± 2.5 h	41.9 ± 1.9 h	58.0 ± 0.6 h
UC-MSCs	32.4 ± 2.2 h	44.8 ± 0.5 h	59.1 ± 1.4 h
DPSCs	27.7 ± 0.8 h	38.8 ± 1.7 h	52.1 ± 1.0 h

An increase in PDT values was observed at later passages in all studied MSC populations, indicating a gradual reduction in proliferative activity during *in vitro* expansion.

All studied MSC populations demonstrated active proliferation and the ability to form colonies under clonal density conditionss. At the same time, changes in proliferative parameters were observed during prolonged *in vitro* cultivation.

### 3.3 Immunophenotyping of MSCs obtained from various tissue sources

The immunophenotype of the studied MSCs was evaluated by flow cytometry (Figure 3; Table 3). All studied MSC populations demonstrated positive expression of CD73, CD90, and CD105 and lacked expression of hematopoietic markers CD34 and CD45, as well as HLA-DR. In DPSCs, nestin expression was additionally evaluated. The obtained immunophenotypic profiles were consistent with the characteristic features of MSCs.

**FIGURE 3:**
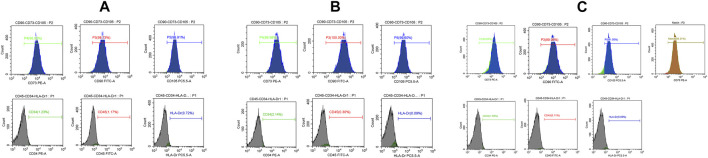
Representative immunophenotypic profile of the ADSCs **(A)**, UC-MSCs **(B)**, and DPSCs **(C)** population.

**TABLE 3:** Immunophenotypic profiles of the ADSCs, UC-MSCs, and DPSCs populations at passage 3 (n = 3, M±SD).

MSC population	СD90	CD105	CD73	Nestin	CD34	CD45	HLA-DR
ADSCs	99.92% ± 2.1%	98.73% ± 1.2%	98.91% ± 0.9%	-	1.23% ± 1.4%	1.17% ± 2.1%	0.72% ± 0.4%
UC-MSCs	99.98% ± 2.6%	100% ± 3.1%	99.60% ± 3.6%	-	2.14% ± 0.9%	0.3% ± 0.1%	0.09% ± 0.01%
DPSCs	99.66% ± 2.7%	99.06 ± 2,1%	91.00% ± 1.18%	98.31% ± 2.4%	1.59% ± 0.01%	0.11% ± 0.02%	0.09% ± 0.09%

### 3.4 Evaluation of adipogenic and osteogenic differentiation potential of MSCs derived from different tissue sources

According to the minimal criteria proposed by the International Society for Cellular Therapy (ISCT), multipotent differentiation capacity is one of the defining characteristics of mesenchymal stromal cells (MSCs) (Dominici et al., 2006). Therefore, the differentiation potential of UC-MSCs, ADSCs, and DPSCs was evaluated by induction toward adipogenic and osteogenic lineages *in vitro*.

Representative images of adipogenic and osteogenic differentiation of MSCs at passage 3 (P3) are presented in Figure 4. The analysis was performed for each donor in triplicate. Following induction, the cultured cells demonstrated morphological changes. Thus, MSCs accumulated intracellular lipid vacuoles during adipogenic inductiona, whereas osteogenic induction resulted in the formation of mineralized extracellular matrix.

**FIGURE 4:**
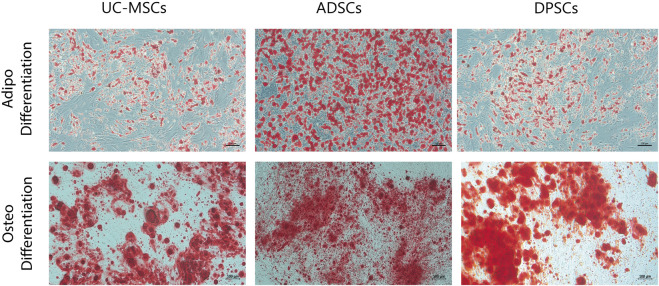
Representative images of cytochemical detection of lipid vacuoles by Oil Red O staining (upper panel) and mineralized matrix deposits by Alizarin Red S staining (lower panel) on day 21 post-induction Phase-contrast and light microscopy. Scale bar = 100 µm.

Thus, studied MSCs derived from adipose tissue, umbilical cord and dental pulp met the minimal criteria for mesenchymal stromal cells demonstrating typical fibroblast-like morphology, high proliferation ability, characteristic phenotype and ability to undergo adipogenic and osteogenic differentiation. This provided the basis for subsequent experiments evaluating the production and physicochemical characteristics of extracellular vesicles under different storage conditions.

### 3.5 Isolation and characterization of extracellular vesicle preparations from conditioned medium

In all experiments, Hank’s solution was used as a negative control. Using NTA, quantitative analysis of extracellular vesicles obtained from UC-MSCs, ADSCs, and DPSCs culture media was performed immediately after sample preparation. The highest yield of EVs was isolated from the conditioned media of UC-MSCs (191 × 10^9^ ± 25 particles/mL) and DPSCs (161 × 10^9^ ± 35 particles/mL), while from the same volume of ADSCs CM was obtained 15,5х10^9^ ± 6,5 particles/mL (Figure 5).

**FIGURE 5:**
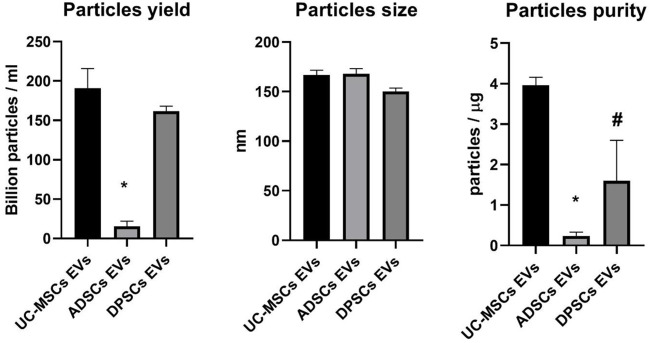
Quantitative characterization of extracellular vesicles derived from UC-MSCs, ADSCs, and DPSCs, including particle yield (left panel), mean particle size (middle panel), and particle purity expressed as particles-to-protein ratio (right panel). Data are presented as mean ± SD. Method: nanoparticle tracking analysis (NTA) and protein quantification by Pierce BCA assay. *p < 0.05 compare to the UC-MSCs EVs and DPSCs EVs. #p < 0.05 compare to the UC-MSCs EVs and ADSCs EVs.

The predominant particle size distribution of UC-MSCs- and DPSCs-derived EVs ranged from approximately 150–170 nm, whereas ADSC-derived EV preparations demonstrated a more heterogeneous size distribution (Figures 5, 6).

**FIGURE 6:**
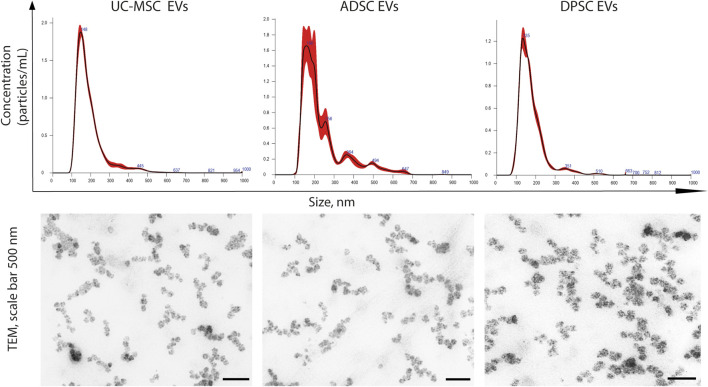
Representative histograms of the quantitative analysis of extracellular vesicles derived from UC-MSCs, ADSCs, and DPSCs. Upper panels–particle size and concentration distributions. Method: nanoparticle tracking analysis (NTA). Lower panel–Representative transmission electron micrograph of a whole-mounted extracellular vesicle preparation stained with uranyl acetate-methyl cellulose. Scale bar, 500 nm.

To verify the structural integrity and morphological characteristics of the isolated EVs, whole-mount transmission electron microscopy (TEM) was performed (Figure 6, lower panel). Ultrastructural examination revealed a high-purity yield of vesicles with diameters predominantly ranging between 100 and 150 nm, consistent with the established size profile of small EVs. Under staining with a uranyl acetate-methyl cellulose mixture, the vesicles exhibited the characteristic cup-shaped (biconcave) morphology typically induced by sample dehydration during whole-mount preparation. The background remained clear of significant proteinaceous aggregates or cellular debris, confirming the efficiency of the isolation protocol. While the majority of EVs were well-dispersed across the Formvar-carbon matrix, minor physiological clustering or artifactual aggregation was observed, as is characteristic for concentrated vesicular suspensions.

Additionally, the protein concentration of each sample was determined the Pierce method (Figure 7). Total protein quantification may provide indirect information regarding the yield, purity and physicochemical characteristics of EV preparations.

**FIGURE 7:**
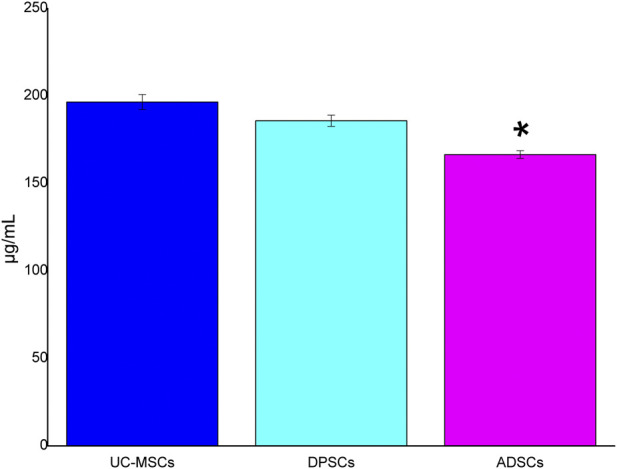
Total amount of protein present in EVs isolated from different types of MSCs (UC-MSCs, DPSCs, ADSCs). Pierce method. (M±SD, n = 3, *p < 0.05 compared to UC-MSCs, DPSCs).

The particle-to-protein ratio indicated the presence of non-vesicular protein components in EV preparations derived from all studied MSCs. However, EV preparations obtained from UC-MSCs demonstrated a comparatively higher particle-to-protein ratio, suggesting greater physicochemical purity relative to the other groups (Figure 5). All EV preparations demonstrated positive expression of the EV-associated markers CD63, CD81, and TSG101 (Figure 8). EV preparations derived from all three MSC sources showed relatively high levels of CD63 and TSG101 and moderate expression of CD81, consistent with the presence of extracellular vesicle-associated proteins. In addition, calnexin was not detected in the analyzed samples, indicating low levels of contamination by intracellular cellular components.

**FIGURE 8:**
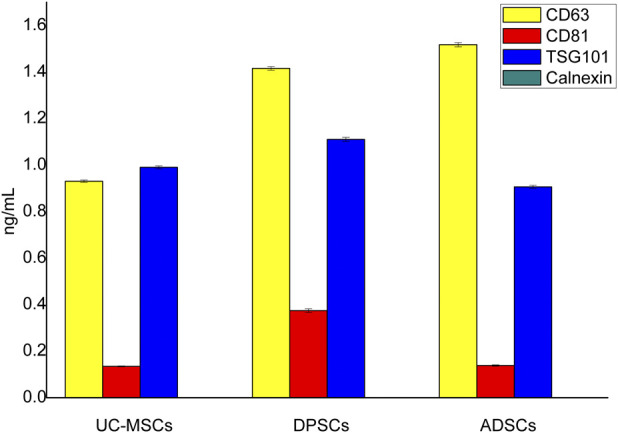
Expression of the CD63, CD81, TSG101 and Calnexin in EVs preparations derived from different types of MSCs [UC-MSCs, DPSCs, ADSCs). (M±SD, n = 3)] Method: ELISA.

Taken together, obtained physicochemical and marker characterization data support the successful isolation of EV preparations from different MSC populations.

### 3.6 Assessment of the stability of EVs from mesenchymal stromal cells under various storage conditions

The stability of EV preparations derived from different MSCs was assessed by evaluating changes in EVs concentration, vesicle size, and total protein concentration over specified time intervals under storage conditions of +4 °C to +6 °C, −20 °C, and −80 °C (Welsh et al., 2024).

Initial physicochemical characteristics were determined immediately after isolation, after which samples from each MSC source were aliquoted into 2 mL portions with concentration 1 × 10^9^ particles/mL for each individual assessment point and stored under the specified temperature conditions for the defined study period (Table 4).

**TABLE 4:** Overall research plan for stability assessment of EVs.

Storage conditions	Time points	EVs type	Evaluation methods
+4 ^°^С - +6 ^°^С	24 h	UC-MSCs derived EVs; ADSCs derived EVs; DPSCs derived EVs	NTA; pierce BCA protein assay
	72 h		
	5 days		
	7 days		
	10 days		
	20 days		
−20 ^°^С	1 month	UC-MSCs derived EVs; ADSCs derived EVs; DPSCs derived EVs	NTA; pierce BCA protein assay
	2 months		
	4 months		
	6 months		
	8 months		
	10 months		
−80 ^°^С	1 month	UC-MSCs derived EVs; ADSCs derived EVs; DPSCs derived EVs	NTA; pierce BCA protein assay
	2 months		
	4 months		
	6 months		
	8 months		
	10 months		

The summary results from three independent experiments are presented in Figures 9, 10; Table 5. Particle concentration in EV preparations remained stable during the first 72 h of storage in saline buffer at +4 °C, but slightly decreased by day 5 of storage. A significant reduction in particle concentration was observed in all EV preparations after 20 days of storage (Figure 9, upper panel). Protein concentration in EVs derived from different MSC populations remained stable for up to 20 days of storage at +4 °C, after which a statistically significant decrease was observed (Figure 9, lower panel). It should be noted that the size distribution of EVs remained unchanged during storage at +4 °C to +6 °C (data not shown). Thus, the physicochemical parameters of EVs derived from MSCs of different tissue origins, specifically particle concentration and total protein concentration, remained stable for at least 14 days when stored at +4 °C to +6 °C.

**FIGURE 9:**
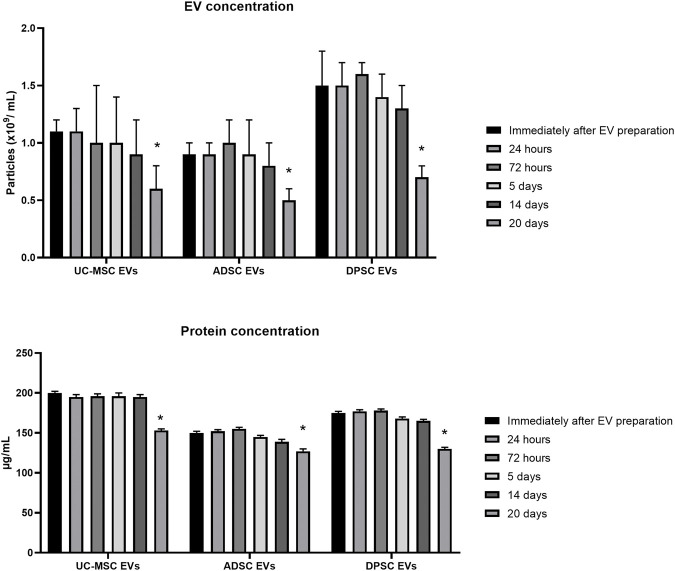
Changes in the physicochemical parameters of EV preparations derived from MSCs of different tissue origins during storage at +4 °C to +6 °C. Data are presented as mean ± SD (n = 3). *p < 0.05 compared with baseline (day 0).

**FIGURE 10:**
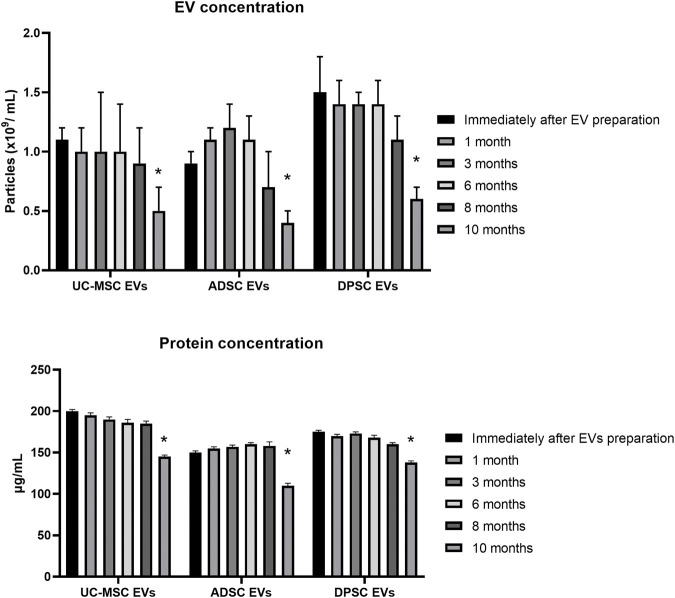
Changes in the physicochemical parameters of EV preparations derived from MSCs of different tissue origins during storage at −20 °C. Data are presented as mean ± SD (n = 3). *p < 0.05 compared with baseline (day 0).

**TABLE 5:** Results of the stability assessment of EVs derived from MSCs of different tissue origins. Storage conditions: −80 °С. (n = 3, M±SD, *р<0.05 compared with the values at the zero point).

EVs type	Storage conditions: −80 °С								
	EVs concentration, bil/mL	EVs size, nm	Protein concentration, µg/mL	EVs concentration, bil/mL	EVs size, nm	Protein concentration, µg/mL	EVs concentration, bil/mL	EVs size, nm	Protein concentration, µg/mL
Initial data obtained immediately after EVs preparation (zero point)				1 month			3 months		
UC-MSCs derived EVs	1.1 ± 0.1	170 ± 2	200 ± 2	1.0 ± 0.2	170 ± 5	195 ± 3	1.0 ± 0.5	162 ± 6	190 ± 3
ADSCs derived EVs	0.9 ± 0.1	168 ± 3	150 ± 2	1.1 ± 0.1	162 ± 3	155 ± 2	1.2 ± 0.2	160 ± 3	157 ± 2
DPSCs derived EVs	1.5 ± 0.3	160 ± 3	175 ± 3	1.4 ± 0.2	160 ± 5	180 ± 2	1.4 ± 0.1	165 ± 5	183 ± 2
6 months				8 months			10 months		
UC-MSCs derived EVs	1.0 ± 0.4	155 ± 3	186 ± 4	0.9 ± 0.3	158 ± 2	185 ± 3	0.8 ± 0.2	160 ± 3	185 ± 2
ADSCs derived EVs	1.1 ± 0.2	165 ± 3	160 ± 2	0.8 ± 0.3	190 ± 4	158 ± 5	0.8 ± 0.1	155 ± 3	155 ± 3
DPSCs derived EVs	1.4 ± 0.2	160 ± 5	178 ± 3	1.3 ± 0.2	168 ± 3	180 ± 2	1.2 ± 0.1	150 ± 2	175 ± 2

During storage at −20 °C, the concentration of EV particles derived from UC-MSCs, ADSCs, and DPSCs remained relatively stable for up to 6 months. A slight decrease in particle concentration was observed after 8 months of storage, while a statistically significant reduction was detected after 10 months in all studied EV preparations (Figure 10, upper panel). Similarly, total protein concentration in EV preparations remained stable during the first 8 months of storage at −20 °C. However, after 10 months, a statistically significant decrease in protein concentration was observed in EVs derived from all studied MSC populations (Figure 10, lower panel). Overall, the obtained results indicate that the selected physicochemical characteristics of MSC-derived EV preparations are largely preserved during long-term storage at −20 °C for at least 6 months.

According to the analyzed parameters, EV preparations derived from MSCs of different tissue origins preserved their physicochemical characteristics for at least 10 months when stored at −80 °C (Table 5).

To provide additional insight into the preservation of the biologically relevant protein cargo of EV preparations during storage, the content of basic fibroblast growth factor (bFGF), a representative bioactive growth factor associated with EV preparations, was additionally evaluated under selected storage conditions (Figure 11). Two-way ANOVA revealed no significant interaction between storage condition and MSC source (P = 0.9409), indicating that the effect of storage on bFGF content was consistent across EVs derived from different MSC sources. A significant effect of MSC source was observed (P = 0.0110), whereas the storage condition itself had no significant effect on bFGF content (P = 0.1640). These findings suggest that the evaluated storage conditions did not substantially affect the preservation of bFGF within the investigated storage period, although further studies are required to determine whether preservation of this growth factor is accompanied by maintenance of overall EV biological activity.

**FIGURE 11:**
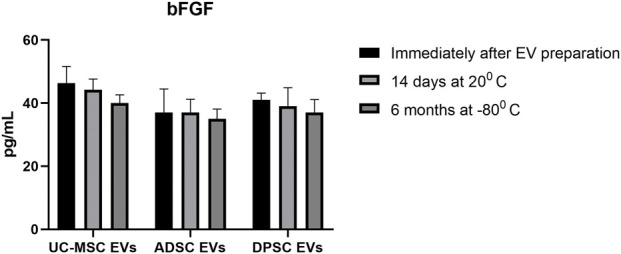
bFGF content in EV preparations immediately after isolation, after 14 days at +20 °C, and after 6 months at −80 °C. ELISA method.

Tacking together, the key physicochemical parameters of EVs derived from UC-MSCs, ADSC and DPSCs as a concentration, size and total protein concentration remained stable for at least 14 days at +4 °C to +6 °C, for at least 6 months at −20 °C and for at least 10 months when stored at −80 °C. The preservation of bFGF may be considered an additional quality-related characteristic of EV preparations, complementing conventional physicochemical parameters.

### 3.7 Discussion

Manufacturing of biological drugs is often associated with substantial variability between different laboratories due to differences in production protocols, raw materials, culture conditions, and analytical methods. Development of EV-based medical products for diagnostic or therapeutic applications is also strongly dependent on the properties of the parental cells, EV isolation methods, cargo composition, storage conditions, and downstream characterization approaches (Adlerz et al., 2020). Successful isolation and characterization of EVs from MSCs conditioned medium first of all require careful standardization and characterization of MSCs cell culture (Welsh et al., 2024). Important experimental parameters include passage number, cell seeding density, culture confluency at the time of conditioned medium collection, volume of culture medium, and specific culture conditions (e.g., oxygen and carbon dioxide concentrations) (Roszkowski, 2024; Kahmini and Shahgaldi, 2021). The complete composition of the culture medium, including the presence of glucose, vitamin supplements, antibiotics, and growth factors, should also should be clearly defined (De Almeida Fuzeta et al., 2020). The use of serum, platelet lysate, or any xenogeneic supplements during EVs collection may introduce exogenous vesicular components and complicate downstream EV characterization (Gowen et al., 2020). In our study, we confirmed that UC-MSCs, ADSCs, and DPSCs met the minimal criteria defining MSCs, while the applied culture conditions allowed successful cell expansion sufficient to obtain therapeutically relevant quantities of EVs. Previously, we demonstrated that MSCs can be maintained under serum-free conditions while preserving their characteristic morpho-functional properties (Gordiienko et al., 2025; Zlatska et al., 2020). Therefore, serum-free culture conditions were used in the present study to minimize contamination from exogenous vesicular components.

According to the recommendations of the ISEV, the development of EV-based products for potential biomedical applications requires a comprehensive quality control strategy that includes characterization of both EV-producing cells and EV preparations (Welsh et al., 2024). Therefore, assessment of MSC characteristics, including viability, proliferative activity, immunophenotype, and differentiation potential, represents an important stage of EV production standardization. Since nanoparticle tracking analysis (NTA) measures the total number and size distribution of nanoparticles in suspension without direct discrimination between membrane-bound vesicles and non-vesicular particles, additional characterization approaches are required for more comprehensive evaluation of EV preparations (Ragni, 2025; Kimiz-Gebologlu and Oncel, 2025; Dave et al., 2025; Lyu et al., 2022; Hwang et al., 2025). In the present study, the particle-to-protein ratio and detection of EV-associated markers were used as complementary physicochemical characterization parameters.

Particular attention should be paid to total protein content, as protein quantification may provide indirect information regarding the yield and physicochemical characteristics of EV preparations. Since the present study focused on long-term storage-associated changes, total protein concentration was used as one of the monitored physicochemical parameters during storage. Changes in protein concentration during prolonged storage may also reflect partial degradation of protein components associated with EV preparations. However, elevated protein levels relative to particle concentration may also reflect the presence of non-vesicular proteins or cellular debris introduced during EV isolation (Ragni, 2025; Kimiz-Gebologlu and Oncel, 2025; Dave et al., 2025; Lyu et al., 2022; Hwang et al., 2025).

Despite the rapidly growing interest in MSC-derived EVs as therapeutic products, there is still no universally accepted standard for EV storage conditions, including temperature, buffer composition, storage duration, and freeze–thaw handling. Published data on EV storage remain highly controversial due to the use of different preservation buffers, including PBS, Hank’s solution, formulations supplemented with BSA, and protocols with or without cryoprotectants (Ahmadian et al., 2024). Most previous studies evaluated EV stability under laboratory research conditions, whereas the present work focused on storage parameters more relevant for future clinical translation of EV-based therapeutics. In our study, EV preparations were stored in Hank’s saline solution without the addition of cryoprotective agents, which may increase the translational applicability of the obtained results and simplify the potential clinical use of EV-based products. Cryoprotective agents such as dimethyl sulfoxide (DMSO), trehalose, glycerol, and human serum albumin are widely investigated for improving preservation of EV physicochemical properties during freezing and long-term storage. These agents may reduce vesicle aggregation, membrane damage, and cargo degradation after freezing and thawing. However, the use of cryoprotectants may complicate further clinical translation of EV-based products, since additional purification steps may be required before administration due to the potential toxicity and adverse effects associated with some cryoprotective compounds, particularly DMSO (Ahmadian et al., 2024). Storage at +4 °C to +6 °C and −20 °C may facilitate handling and short-to medium-term preservation of EV preparations using conventional laboratory refrigeration systems. Such conditions could simplify the implementation of EV-based products in research and clinical environments where specialized cryogenic infrastructure is limited (Ragni, 2025; Kimiz-Gebologlu and Oncel, 2025). In contrast, storage at −80 °C requires specialized cryogenic equipment. However, the obtained results suggest that these conditions may provide improved preservation of selected physicochemical characteristics of EV preparations during long-term storage and transportation between research, manufacturing, and clinical facilities (Hwang et al., 2025; Guarro et al., 2022; Yuan et al., 2021).

The novelty of the present study lies not only in the evaluation of EV stability under different temperature conditions, but also in the comparative analysis of EVs derived from multiple MSC sources stored in clinically applicable saline-based formulations. Different tissue-derived MSC-EVs may possess distinct membrane composition and cargo profiles, potentially affecting their storage stability and sensitivity to freezing conditions.

In addition to the physicochemical characterization of EV preparations, we evaluated the content of basic fibroblast growth factor (bFGF) as a representative biologically active protein associated with EV preparations. Preservation of specific bioactive molecules during storage may provide complementary information beyond particle concentration, size distribution, and total protein content, particularly because therapeutic effects of MSC-derived EVs are largely mediated by their molecular cargo. In the present study, bFGF content remained comparable under the evaluated storage conditions, and storage condition had no significant effect on its concentration. These findings suggest that, under the investigated conditions, storage did not substantially affect the preservation of this selected bioactive protein. Nevertheless, the preservation of a single growth factor should not be interpreted as evidence of complete maintenance of EV biological functionality or overall cargo integrity. The biological activity of EVs results from the combined action of numerous proteins, lipids, and nucleic acids, as well as their interactions with recipient cells. Therefore, although the present findings provide additional evidence supporting the preservation of a representative bioactive protein during storage, dedicated functional assays are required to determine whether these storage conditions also preserve the therapeutic potential of EVs.

Storage of EV preparations in liquid form at low temperatures offers several practical advantages, including simplified sample handling, reduced preparation time before use, and preservation of vesicles without additional processing steps such as lyophilization. However, prolonged storage in liquid conditions may also be associated with several limitations, including vesicle aggregation, alterations in membrane integrity, protein degradation, and potential loss of biologically active cargo during freezing and thawing processes. In addition, repeated freeze–thaw cycles may affect the physicochemical characteristics and recovery of EV preparations. Therefore, optimization of storage conditions remains an important aspect of EV standardization and quality control (Ahmadian et al., 2024; Sivanantham and Jin, 2022; Görgens et al., 2022; Gelibter et al., 2022; Rama Varma et al., 2026).

Up to now, two main approaches for EV preservation have been used: storage in solution and lyophilization (Görgens et al., 2022; Gelibter et al., 2022). An additional limitation of the present study is that EV preparations were evaluated exclusively in liquid form. Lyophilized EV formulations are considered advantageous for long-term storage and transportation due to improved stability and reduced dependence on cold-chain logistics. However, the lyophilization process may induce vesicle aggregation, membrane damage, or alterations in biological cargo without the use of appropriate cryoprotectants (Hwang et al., 2025; Görgens et al., 2022; Gelibter et al., 2022). In contrast, EVs stored in saline solution preserve their native hydrated state and can be directly applied without additional reconstitution procedures, making such formulations more practical for routine clinical use and immediate therapeutic application (Hwang et al., 2025; Guarro et al., 2022). At the same time, similarly to lyophilization, storage of EVs in solution also has several limitations, including the risk of vesicle aggregation, membrane destabilization, and gradual degradation of proteins, lipids, and RNA cargo during long-term storage. In addition, repeated freeze–thaw cycles may significantly affect EV integrity and biological activity. Liquid formulations are also highly dependent on continuous cold-chain maintenance, which may complicate transportation and large-scale clinical application compared to lyophilized products (Yuan et al., 2021).

Taken together, the obtained results provide additional information regarding the preservation of selected physicochemical characteristics of MSC-derived EV preparations under different storage conditions and may contribute to further optimization and standardization of EV handling protocols for research and potential biomedical applications.

## 4 Conclusion

MSCs derived from adipose tissue, umbilical cord, and dental pulp demonstrated characteristic mesenchymal stromal cell properties and differed in the quantity of EV preparations obtained from the same initial culture volume. The particle-to-protein ratio, particle size distribution, and detection of EV-associated markers CD63, CD81, and TSG101 were consistent with EV-enriched preparations derived from all studied MSC populations.

The key physicochemical parameters of EVs derived from UC-MSCs, ADSC and DPSCs as a concentration, size and total protein concentration remained stable for at least 14 days at +4 °C to +6 °C and for at least 6 months at −20 °C. In addition, EV samples preserved their characteristics for at least 10 months when stored at −80 °C. The assessment of bFGF complements conventional physicochemical characterization by providing information on the preservation of a selected biologically relevant quality attribute of EV preparations.

The obtained findings contribute to the growing need for standardized EV storage approaches and may serve as a basis for further studies focused on optimization, reproducibility, biobanking, and potential clinical translation of EV-based products stored in liquid saline formulations.

## Data Availability

The raw data supporting the conclusions of this article will be made available by the authors, without undue reservation.
